# Our plastic age

**DOI:** 10.1098/rstb.2009.0054

**Published:** 2009-07-27

**Authors:** Richard C. Thompson, Shanna H. Swan, Charles J. Moore, Frederick S. vom Saal

**Affiliations:** 1 Marine Biology and Ecology Research Centre, Marine Institute, University of Plymouth, Drake Circus, Plymouth PL4 8AA, UK; 2 Department of Obstetrics and Gynecology, University of Rochester, Rochester, NY, USA; 3 Algalita Marine Research Foundation, CA, USA; 4 Division of Biological Sciences, University of Missouri, MO, USA

**Keywords:** plastic, polymer, debris, endocrine disruption, phthalates, waste management

## Abstract

Within the last few decades, plastics have revolutionized our daily lives. Globally we use in excess of 260 million tonnes of plastic per annum, accounting for approximately 8 per cent of world oil production. In this Theme Issue of *Philosophical Transactions of the Royal Society*, we describe current and future trends in usage, together with the many benefits that plastics bring to society. At the same time, we examine the environmental consequences resulting from the accumulation of waste plastic, the effects of plastic debris on wildlife and concerns for human health that arise from the production, usage and disposal of plastics. Finally, we consider some possible solutions to these problems together with the research and policy priorities necessary for their implementation.

The term plastics applies to a wide range of materials that at some stage in manufacture are capable of flow such that they can be extruded, moulded, cast, spun or applied as a coating. Synthetic polymers are typically prepared by polymerization of monomers derived from oil or gas, and plastics are usually made from these by addition of various chemical additives. There are currently some 20 different groups of plastics, each with numerous grades and varieties (APME 2006). Plastics are incredibly versatile materials; they are inexpensive, lightweight, strong, durable, corrosion-resistant, with high thermal and electrical insulation properties. The diversity of polymers and the versatility of their properties facilitate the production of a vast array of plastic products that bring technological advances, energy savings and numerous other societal benefits (Andrady & Neal 2009). The first truly synthetic polymer, *Bakelite*, was developed by Belgian chemist Leo Baekeland in 1907, and many other plastics were subsequently developed over the next few decades. It was not until the 1940s and 1950s, however, that mass production of everyday plastic items really commenced. On the opening page of their book ‘*Plastics*’, Yarsley & Couzens (1945; first published in 1941) consider that ‘*the possible applications* [of plastics] *are almost inexhaustible*’. At that time, global production was less than a million tonnes per annum, but plastics were already widely used in products ranging from cups and saucers to components for cars and aeroplanes. The final chapter of their book anticipates the ways that plastics will influence the life of someone born 70 years ago at the start of our ‘plastic age’. (Yarsley & Couzens 1945)

This [imaginary] plastic man will come into a world of colour and bright shining surfaces where childish hands find nothing to break, no sharp edges, or corners to cut or graze, no crevices to harbour dirt or germs … . The walls of his nursery, his bath … all his toys, his cot, the moulded light perambulator in which he takes the air, the teething ring he bites, the unbreakable bottle he feeds from [all plastic]. As he grows he cleans his teeth and brushes his hair with plastic brushes, clothes himself with in plastic clothes, writes his first lesson with a plastic pen and does his lessons in a book bound with plastic. The windows of his school curtained with plastic cloth entirely grease- and dirt-proof … and the frames, like those of his house are of moulded plastic, light and easy to open never requiring any paint.

(Yarsley & Couzens 1945, p.149)

The text continues through extensive use of plastics for furniture and interior design, for beauty and leisure, in industry and in transport by road, sea and air. Until in old age plastic man:

wears a denture with silent plastic teeth and spectacles with plastic lenses … until at last he sinks into his grave in a hygienically enclosed plastic coffin

(Yarsley & Couzens 1945, p.152)

This Theme Issue explores the evidence and the diversity of scientific opinion surrounding our use of plastics at the start of the twenty-first century. To set the present day perspective into context, we have included a historical overview summarizing the development and production of plastic, together with associated concerns, regulatory measures and some potential future trends (figure 1). Many of these topics are considered in detail within the Theme Issue, and we have included a selection of quotes from these papers to illustrate the diversity of subject matter, scientific opinions and conclusions therein (table 1).

**Figure 1.**
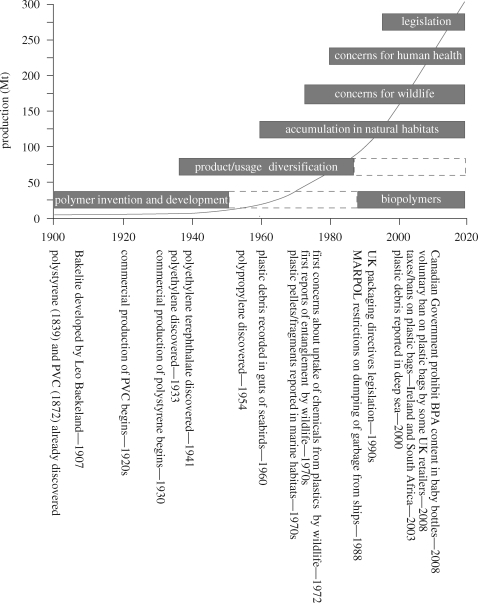
Summary illustrating historical stages in the development, production and use of plastics together with associated concerns and legislative measures (numerous sources). Solid red line shows plastic production in millions of tonnes (Mt). Reproduced with permission from APME (2006). BPA, bisphenol A; PVC, polyvinyl chloride.

**Table 1.** Selected quotes reflecting the diversity of content and some of the scientific conclusions of authors in this Theme Issue. Phthalates, BPA, PBDE and tetrabromobisphenol A (TTBPA) are chemical additives, and in the case of BPA, a monomer used in the production of plastics.

‘Any future scenario where plastics do not play an increasingly important role in human life therefore seems unrealistic’ (Andrady & Neal 2009).
‘One of the most ubiquitous and long-lasting recent changes to the surface of our planet is the accumulation and fragmentation of plastics’ (Barnes *et al.* 2009).
‘Monitoring is crucial to assess the efficacy of measures implemented to reduce the abundance of plastic debris, but it is complicated by large spatial and temporal heterogeneity in the amounts of plastic debris and by our limited understanding of the pathways followed by plastic debris and its long-term fate’ (Ryan *et al.* 2009).
‘The environmental, cultural, aesthetic, commercial and other problems arising from pelagic plastics in particular and varied marine debris items in general are manifold, widely acknowledged and often difficult to address’ (Gregory 2009).
‘As plastics production and usage continue to increase, particularly in economically developing countries, the environmental implications of their disposal should be carefully considered to avoid inadvertent release, magnification and transport of contaminants’ (Teuten *et al.* 2009).
‘Phthalates and BPA have been shown to affect reproduction in all studied animal groups, to impair development in crustaceans and amphibians and to induce genetic aberrations. Molluscs, crustaceans and amphibians appear to be especially sensitive to these compounds, and biological effects are observed at environmentally relevant exposures in the low ng l^−1^ to µg l^−1^ range’ (Oehlmann *et al.* 2009).
‘PBDE and TTBPA have been shown to disrupt thyroid hormone homeostasis while PBDEs also exhibit anti-androgen action. Experimental investigations in animals indicate a wide variety of effects associated with exposure to these compounds, causing concern regarding potential risk to human health’ (Talsness *et al.* 2009).
‘Studies also are needed to identify the phthalate metabolites and BPA species relevant to human health, paying special attention to potentially vulnerable segments of the population (e.g. children, women of reproductive age, minorities)’ (Koch & Calafat 2009).
‘… small changes in hormone levels resulting from exposure may be of public health importance when considering the prevalence of exposure to plastic additives and endocrine disrupting compounds among entire populations’ (Meeker *et al.* 2009).
‘Around 4 per cent of world oil and gas production, a non-renewable resource, is used as feedstock for plastics and a further 3–4% is expended to provide energy for their manufacture. A major portion of plastic produced each year is used to make disposable items of packaging or other short-lived products that are discarded within a year of manufacture. These two observations alone indicate that our current use of plastics is not sustainable. In addition, because of the durability of the polymers involved, substantial quantities of discarded end-of-life plastics are accumulating as debris in landfills and in natural habitats worldwide. Recycling is one of the most important actions currently available to reduce these impacts and represents one of the most dynamic areas in the plastics industry today. Recycling provides opportunities to reduce oil usage, carbon dioxide emissions and the quantities of waste requiring disposal’ (Hopewell *et al.* 2009).
‘Bioplastic polymers have great potential to contribute to material recovery, reduction of landfill and use of renewable resources. Widespread public awareness of these materials and effective infrastructure for stringent control of certification, collection, separation and composting will be crucial to obtaining these benefits in full’ (Song *et al.* 2009).
‘… there is an opportunity to address many of these issues simultaneously by using the science in this issue to help develop an enhanced Road Map for policy around plastics, the environment and human health in the UK’ (Shaxson 2009).
‘… plastic production continues to grow at approximately 9 per cent per annum … . As a consequence, the quantity of plastics produced in the first 10 years of the current century will approach the total that was produced in the entire century that preceded.’ (Thompson *et al.* 2009).

The series of papers starts with a review of the history of polymer development together with some of their applications, past, present and future (Andrady & Neal 2009). This includes the use of lightweight plastic components in cars and aeroplanes to reduce fuel usage; the use of inexpensive plastic casings to make information technology and electrical goods far more readily accessible than would otherwise have been possible; and the use of plastics for sterile dressings and medical products. The most substantial use of plastics today, accounting for well over a third of production, is, however, for disposable items of packaging, most of which are discarded within a year or so of manufacture (Barnes *et al.* 2009; Hopewell *et al.* 2009).

The durability and increasing usage of plastics create a major waste management problem with plastic accounting for approximately 10 per cent of the waste we generate. Some of this is recycled, but a substantial proportion is disposed of to landfill (Barnes *et al.* 2009; Hopewell *et al.* 2009). A range of terms are used to describe the waste that is produced by modern society, these include trash, garbage, rubbish, litter and debris; usage varies according to the type and origin of the waste and according to regional differences in terminology. Usage of these terms is considered to be interchangeable in the papers within this volume; the reader should focus on the types, sources, accumulation, disposal and effects of the waste.

Plastic debris has accumulated in natural habitats from the poles to the equator (Barnes *et al.* 2009); it is a very conspicuous component of the debris that is present in the marine environment, and most of the literature on the accumulation of plastic in the environment and the associated problems for wildlife has come from marine habitats (Gregory 2009). Monitoring represents an important step towards quantifying spatial and temporal trends in the abundance of all types of debris, including plastic. Numerous national and international schemes have been initiated to record quantities and categories (uses, sources, material types, sizes), and in some cases to facilitate debris removal (Ryan *et al.* 2009).

Substantial quantities of plastic debris already contaminate marine habitats from remote shorelines and inaccessible areas of the deep sea to heavily populated coastlines. The ubiquity of this debris in the marine environment has resulted in numerous accounts of species ingesting and becoming entangled in plastic. As a consequence of the durability of plastics, these encounters typically result in injury or impaired movement and can ultimately result in death (Gregory 2009). There is evidence that plastics are fragmenting in the environment and, as a consequence, will become available for ingestion by a wider range of organisms (Barnes *et al.* 2009). In addition to these physical effects, there has been speculation for over 30 years that the ingestion of plastic debris could lead to the transfer of toxic chemicals to wildlife. Recent publications have raised new concerns around this issue (Mato *et al.* 2001; Thompson *et al.* 2004; Arthur *et al.* 2009); Teuten *et al.* (2009) present a summary of current evidence together with new data on the accumulation of chemicals from plastic by wildlife.

In parallel with concerns for wildlife, there is a rapidly growing body of evidence relating to public health issues arising from current use of plastics. A range of chemicals are added to plastics during manufacture, to enhance the performance of plastics. These additives can be referred to as plasticizers and include flame retardants, stabilizers, antioxidants and other chemicals such as antimicrobials that give each type of plastic unique properties. There is concern that potentially harmful chemical additives including phthalates, bisphenol A (BPA) and polybrominated diphenyl ethers (PBDE) could be transferred to humans directly from plastics, for example from flexible toys mouthed by toddlers, or indirectly, for example via food and drink that is packaged or transported via tubing in plastics containing these additives (e.g. Wagner & Oehlmann 2009). Evidence relating to this is considered from three perspectives: human body burdens of chemicals used in plastic manufacture (Koch & Calafat 2009); experimental studies on animals (Talsness *et al.* 2009) and the effects of these chemicals on humans (Meeker *et al.* 2009). These papers present stark evidence and consider some possible solutions.

Looking to the next few decades, it seems inevitable that humankind will become more reliant on plastics; therefore, the Theme Issue examines potential solutions for waste management of used plastics. For packaging applications, in particular, biodegradable plastics have been advocated as an approach that uses renewable biomass and/or facilitates decomposition at the end of a product's lifetime. Song *et al.* (2009) consider the potential applications and subsequent degradation of these materials, presenting new data on biodegradation in domestic composting conditions. The ‘three Rs’ (reduce, reuse and recycle) have been, extensively and lyrically (Johnson 2006; the three Rs: from the album Sing-A-Longs and Lullabies for the Film Curious Ge), advocated as solutions to the wasteful nature of our society. These strategies together with a ‘fourth and fifth R’ (‘energy recovery’ and ‘molecular redesign’) are considered by Hopewell *et al.* (2009) and Thompson *et al.* (2009), who describe current trends and examine the limitations to recycling of plastics.

Having considered the benefits of plastics, the problems associated with production and the use of plastics past and present and some solutions, the Theme Issue also examines the science–policy interface where appropriate directions will be determined by governments and where policies will be implemented to restrict activities and incentivize change (Shaxson 2009). Finally, the guest editors and contributors synthesize the work presented in the Theme Issue as a whole to give a summary of current understanding together with priorities for research, innovation and policy that are required to guide our future use of plastics in relation to the environment and human health (Thompson *et al.* 2009).
